# On the Identification and Use of Social versus Nonsocial Reinforcers: A Review of Research Practices

**DOI:** 10.1007/s40614-024-00426-0

**Published:** 2024-10-29

**Authors:** Samuel L. Morris, Katherine G. Bridges

**Affiliations:** Department of Psychology, Louisiana State University, Baton Rouge, LA USA

**Keywords:** Current practices, Reinforcer identification, Reinforcer type, Preference assessment, Social stimuli

## Abstract

Recent research has developed efficacious methods for identifying individualized social reinforcers and utilizing social reinforcers may be beneficial for several reasons. However, the relative likelihood of utilizing social versus nonsocial reinforcers in behavior-analytic research remains unclear. The aim of this study was to evaluate how likely behavior analysts are to employ social versus nonsocial reinforcers in the context of research. We pursued this aim by evaluating the types of reinforcers utilized in research published during the past 9 years in five applied behavior-analytic journals. Results suggest that researchers in applied behavior analysis have been more likely to use individualized nonsocial reinforcers than individualized social reinforcers. Moreover, when social reinforcers were employed, they were much more likely to be generic and not individualized. These data suggest there is room for improvement in the types of programmed reinforcement contingencies we use in research and ways of facilitating such improvement are discussed. Implications and directions for future research evaluating current practices, demonstrating the utility of social reinforcers, and comparing the utility of social versus nonsocial reinforcers are also discussed.

**Supplementary Information:**

The online version contains supplementary material available at 10.1007/s40614-024-00426-0.

Behavior adapts and develops as it interacts with the environment and contacts differential consequences. In some cases, this process may seem to occur “naturally” as a product of contingencies that are built-in, relatively consistent characteristics of our physical or social environment. However, in other cases artificial, programmed reinforcement contingencies are needed to facilitate behavior change. When behavior analysts employ artificial reinforcers, several choices must be made that can affect the efficacy and feasibility of the programmed contingency as well as the extent to which it facilitates generalization and maintenance of behavior change. Among other choices, behavior analysts must decide what type of reinforcers they will utilize. Commonly programmed artificial reinforcers may be categorized into three general types: edible reinforcers (i.e., tangible, consumable stimuli, e.g., a cookie), leisure reinforcers (i.e., tangible, nonconsumable stimuli, e.g., crayon and paper, videos on a tablet), and social reinforcers (i.e., nontangible stimuli delivered by one person to a another or interaction between two or more individuals, e.g., praise, conversation, hide-and-seek).^1^ In the past, research on methods of reinforcer identification has focused on evaluating edible and leisure reinforcers (e.g., DeLeon & Iwata, 1996; Fisher et al., 1992; Hanley et al., 2003; Roane et al., 1998). However, the identification and use of individualized social reinforcers in addition to nonsocial reinforcers may afford several benefits.

Social reinforcers can allow for more choice and reinforcer variability (e.g., Egel, 1981; Hanratty & Hanley, 2021), are practical to deliver and readily available in the natural environment (DeLeon et al., 2013), such that they may facilitate maintenance and generalization (Koegel & Rincover, 1977; Stokes & Baer, 1977), may avoid the adverse health effects of reliance on calorically dense edible items or sedentary leisure activities as reinforcers (e.g., Chen et al., 2016; Must et al., 2014), and may be especially helpful in evaluating and improving social behavior (e.g., Kronfli et al., 2023; Morris & Vollmer, 2022a).^2^ All of these benefits may increase the efficacy of a variety of behavior change procedures that are commonly used in clinical practice and research. Just as important, the use of individualized social reinforcers may improve the social validity, acceptability, and adoption of behavioral interventions relative to those built around the contingent delivery of some types of edible (e.g., candy) or leisure (e.g., screen-based media) reinforcers. Finally, the identification and use of individualized social reinforcers can help behavior analysts fulfill their ethical obligation to individualize procedures and avoid potentially harmful reinforcers (Behavior Analyst Certification Board, 2020, Guidelines 2.09, 2.13, 2.14, 2.15, 3.01). See Morris et al. (2023c) for an extended discussion of the difficulties and benefits of identifying individualized social reinforcers.

Perhaps these benefits are, at least in part, what led DeLeon et al. (2013) to recommend that clinicians and researchers first attempt to identify or establish social reinforcers prior to identifying and employing leisure or edible reinforcers (see their Fig. 3.1). Since their recommendation, a body of research focused on evaluating and improving methods of identifying individualized social reinforcers has emerged (Clay et al., 2013, 2018; Davis et al., 2017, 2022; Harper et al., 2021; Huntington & Higbee, 2018; Huntington & Schwartz, 2022; Kelly et al., 2014; Kronfli et al., 2023; Lang et al., 2014; Morris & Vollmer, 2019, 2020a, 2020b, 2020c; Morris et al., 2023a; Nuernberger et al., 2012; Wolfe et al., 2018). A recent review synthesizing this available evidence suggests that research on methods of social preference assessment has yielded an effective technology for identifying individualized social reinforcers and discriminating their relative efficacy (Morris et al., 2023c). Given the utility of social reinforcers and the development of effective and efficient methods for identifying them, it is important to consider how likely behavior analysts are to utilize social versus nonsocial reinforcers in clinical practice and research.

Graff and Karsten (2012) surveyed clinicians providing behavior-analytic services to better understand how they identify and use reinforcers. Their results indicated that clinicians were more likely to use informal (i.e., casual observation, asking the client) or indirect (i.e., asking others) methods of reinforcer identification than formal methods of preference assessment. Many clinicians reported using methods of formal preference assessments, but such methods were employed much less frequently than informal or indirect methods. Graff and Karsten also asked respondents whether they utilized different types of reinforcers. Of the behavior analyst surveyed, they found that 90% of their respondents reported utilizing some form of social stimuli, 69% reported using edible stimuli, and 71% reported using leisure stimuli as a part of programmed reinforcement contingencies.

Morris et al. (2024) provided an updated description of the identification and use of social versus nonsocial reinforcers in clinical practice. In addition to evaluating whether or not different types of stimuli were ever programmed as reinforcers and how different types of reinforcers were identified, Morris et al. extended Graff and Karsten (2012) by surveying clinicians about (1) their relative probability of using each type of reinforcer (i.e., not just whether it is ever used, but what percentage of the time) and (2) how often social reinforcers are individualized to the client versus generic or consistent across clients. Results indicated that most respondents employ all three types of reinforcers to some extent, with fewer using edible (i.e., 86%) than leisure (i.e., 99%) or social (i.e., 97%) reinforcers. Regarding the relative likelihood of using different types of reinforcers, respondents reported most frequently using leisure reinforcers, followed by social reinforcers, and then edible reinforcers in practice. Finally, respondents indicated that when social stimuli were incorporated into programmed reinforcement contingencies, they were sometimes generic but usually individualized.

Although many descriptions of clinical practices related to reinforcer identification exists (e.g., Graff & Karsten, 2012; Morris et al., 2023b, 2024) the details of how different types of reinforcers are identified and used in research are unknown. The different contingencies operating on the behavior of researchers and clinicians may lead to different practices related to the identification and use of different types of reinforcers. For example, the practical constraints of clinical practice may favor efficient methods of identifying reinforcers which can be easily and frequently delivered. In contrast, the requirements of research may favor more formal, data-based methods of identification and the use of reinforcers that are maximally efficacious. However, methods of identification and types of reinforcers employed may be more individualized in clinical practice, whereas these procedures may be dictated by the specific empirical question being evaluated in a research context. Thus, further investigation is needed to evaluate current practices related to identifying and using different types of reinforcers in research and how they may differ from clinical practice. Evaluating the reinforcement procedures employed in applied, behavior-analytic research may identify gaps in our extant evidence base and elucidate potential variables influencing the efficacy, effectiveness, and adoption of behavior-change procedures. Moreover, differences in reinforcement procedures between research and practice may inform future research on methods of identifying different types of reinforcers that could serve to empirically evaluate and resolve disconnects between research and clinical practice.

In summary, behavior analysts may often use artificial, programmed reinforcers to bring about behavior change when contingencies available in the natural environment are insufficient. Determining what type of reinforcer to use is an essential part of this process because, outside of the context of function-based interventions, edible, leisure, and social reinforcers may be equally viable options. However, social reinforcers may offer several benefits over nonsocial reinforcers. Recent research suggests we have an effective technology for identifying individualized social reinforcers and discriminating their relative efficacy (Morris et al., 2023c). However, it remains unclear how likely behavior analysts are to employ different types of reinforcers. Thus, the purpose of the current study was to evaluate the identification and use of social versus nonsocial reinforcers in behavior-analytic research. In particular, we reviewed a sample of 2,429 behavior-analytic research articles to ascertain the relative likelihood with which researchers (1) use preference assessments to identify individualized edible, leisure, versus social reinforcers and (2) incorporate individualized, generic, versus no social consequences as a part of their procedures. A secondary aim was to investigate how the relative likelihood of using different types of reinforcers or incorporating different types of social stimuli varied based on different characteristics of the article’s statement of purpose.

## Method

We reviewed articles published across five journals: *Behavior Analysis in Practice, Behavior Analysis: Research and Practice, Behavioral Interventions*, *Behavior Modification*, and the *Journal of Applied Behavior Analysis*. We reviewed each and every article published by these journals between 2015 and 2023. We selected these journals because they frequently publish applied, behavior-analytic research, because we anticipated that a high proportion of the articles published would incorporate stimulus preference assessments, and because each had recently published research related to methods of assessing preference for social stimuli. However, it should be noted that this is not an exhaustive list of journals meeting these criteria and the exclusion of articles published in journals outside of those above is a limitation of the current review. We reviewed all articles from the nine most recent years to obtain a current picture of research practices, but also obtain a large enough sample to capture changes across time. In addition, several early methods of assessing preference for social interaction were published between 2012 and 2014 (e.g., Clay et al., 2013; Kelly et al., 2014; Lang et al., 2014; Nuernberger et al., 2012); thus, 2015 served as a good starting point. In total, 2,429 articles were included in this review.

### Coders and Initial Training

Graduate students in a behavior-analytic masters or PhD program and undergraduate students who had taken at least one course related to applied behavior analysis served as coders. Coders were trained to utilize the coding system using a set of 10 articles that were not included in the review (i.e., were published prior to the first year included). Training consisted of a didactic introduction to each component of the coding system, including precise instructions, operational definitions, and examples and nonexamples for each variable coded. Then, coders attempted to independently utilize the coding system on the training articles and received feedback on their performance after completing each article. Once trainees’ coding of the training articles yielded 100% agreement with that of the first or second author, they began coding articles to be included in this review.

### Dependent Measures

For each article, we recorded the title as well as the journal, year, and issue in which it was published. We also coded the following five characteristics of each article based on the authors’ description. First, we determined whether each article included original data obtained from human participants. Coders searched^3^ for the word “method” within all articles, then reviewed all instances of the word. Articles that did not contain a method section were likely a discussion, technical article, or commentary, and did not fit our criteria to be coded as original data. If the article did not contain a method section, it was coded as not including original data. Next, coders searched for the terms “purpose” and “aim” and reviewed all instances of these words in the article. Articles in which the authors described the purpose as reviewing or synthesizing previous research, conducting secondary or meta-analyses of previously published data, or providing a discussion or commentary were coded as not including original data. In addition, as an artifact of our specific research question, all articles including only nonhuman animals as participants were coded as not including original data. All articles not meeting any of the above exclusion criteria were coded as including original data.

Second, for all articles including original data, we evaluated whether researchers individualized their reinforcement procedures by conducting a stimulus preference assessment. Preference assessments are the most ubiquitous and evidence-based method for making data-based decisions about which stimuli are likely to function as reinforcers for a given individual (Hagopian et al., 2004; Morris et al., 2023c; Tullis et al., 2011). Thus, the inclusion of a preference assessment was used as a criterion for whether or not a study included individualized reinforcers. Although this criterion allowed us to readily, consistently analyze a large number of articles, it also limits the scope and clarity of our conclusions (see “Discussion”). We coded whether a study conducted a stimulus preference assessment by searching for the term “pref...” and reviewing all instances of the word in each article. Articles were coded as including a stimulus preference assessment if the authors described conducting a preference assessment with (e.g., “... a paired-stimulus preference assessment was conducted...”) or assessing preferences of their participants (e.g., “... we assessed participants’ preference for different leisure items using a paired-stimulus preference assessment...”). If the authors of the articles indicated in any way that a preference assessment was used to assess a participant's preference between two or more stimuli, then they were coded as including a preference assessment. Articles that described the preference assessment as being conducted “previously” or “prior to the experiment proper” were still coded as including a preference assessment. Articles that did not include such descriptions or only stated that they included preferred stimuli or individualized reinforcers without specifying how they were identified were coded as not including a preference assessment (e.g., “highly preferred leisure items were provided as a consequence”).^4^

Third, for all articles that included a preference assessment, we coded the type of stimuli evaluated during the preference assessment. We coded the type of stimuli evaluated in each preference assessment by searching for the term “pref...” and reviewing all sections of the article in which the term occurred. Coders were instructed to begin this search in the part of the method section that was previously identified as describing that a preference assessment was conducted. Articles that described evaluating “edible” stimuli or two or more tangible, consumable stimuli (e.g., candy, food, drink) were coded as evaluating edible stimuli. Articles that described evaluating “leisure” stimuli or two or more tangible, nonconsumable stimuli (e.g., toy cars, blocks) or activities (e.g., watching videos or music on a device) were coded as evaluating leisure stimuli. Articles that described evaluating “social” stimuli, “social interaction,” “attention,” or two or more types of nontangible, social stimuli delivered from one individual to another (e.g., praise, tickles) or two or more types of interaction between two or more people (e.g., conversation, dancing) were coded as evaluating social stimuli. Multiple stimulus types could be evaluated in a single article. For example, if an article included two types of stimuli within a preference assessment or across multiple preference assessments, then both were coded as being evaluated in that article. Articles evaluating stimuli (e.g., colors; Hardesty et al., 2023) or activities (e.g., intervention strategies; Peltier et al., 2023) that could not be categorized as edible, leisure, or social stimuli were coded as not including edible, leisure, or social stimuli in the preference assessment.

Fourth, for all articles that included original data, we evaluated whether any type of social stimuli was included as a programmed consequence during the assessment or intervention sessions conducted. In particular, we reviewed the method section of each article to determine whether any form of social consequence (e.g., attention, praise, social interaction or play) was incorporated. Coders searched for the terms “attention,” “play,” “interaction,” “praise,” and “social.” For each article, all instances of each of these words were reviewed. Instances in which these terms were used to describe programmed consequences in an article’s method section were of primary interest for this analysis. Articles that included some form of programmed social consequences using any of the terms listed above but did not individualize the social consequences based on the results of a preference assessment, were characterized as including generic or nonindividualized social consequences (e.g., praise or attention delivered in a similar or unspecified way to all participants). Articles that did not specify the inclusion of any type of programmed social consequences using any of the terms listed above were coded as including no social consequences.

Fifth, to investigate variables that may influence the use of different types of individualized reinforcers and the use of different types of social stimuli, we coded characteristics of the purpose of all articles that conducted a preference assessment. For all articles that included both original data and conducted a preference assessment, we first evaluated if the purpose of the article was to evaluate or improve the methods of preference assessment. Coders searched for the terms “purpose,” “aim,” and “study” and reviewed all instances in each article. If the purpose of the article indicated that its primary focus was to improve or evaluate methods of preference assessment, then the article was coded as a “preference assessment study” and no additional purpose characteristics of that article were coded. If purpose of the article remained unclear after this initial step, then coders searched for the terms “pref...,” “SPA,” and “PA” and reviewed all instances of these terms. If the article described conducting a preference assessment and also described a statement of purpose beyond that of evaluating or improving preference assessment methodology, then the article was coded as a “non-preference assessment study” and additional characteristics of the purpose of that article were investigated in subsequent analyses.

For articles that included original data and conducted a preference assessment but did not include a statement of purpose related to evaluating or improving preference assessment methodology, we also coded two other characteristics of the statement of purpose. One characteristic that was coded was whether the article focused on intervention strategies or assessment strategies. This characteristic was coded by searching for the terms “purpose,” “aim,” and “study” and reviewing all instances of each to identify statements of purpose. If an article included a statement of purpose that incorporated terms such as “interven...,” “change,” “modify,” or “influence,” then the article was coded as having aims related to intervention strategies. If an article included a statement of purpose that did not incorporate any of the intervention-related terms described above, then that article was coded as having aims related to assessment strategies. The coding of aims related to intervention versus assessment strategies was mutually exclusive: an article either included intervention-related terms in their statement of purpose and was coded as an “intervention study” or did not include such terms and was coded as an “assessment study.” In cases that were unclear, coders were instructed to read the entirety of the last paragraph of the introduction section and first paragraph of the discussion section prior to determining what to code.

The other characteristic that was coded was whether the article included a statement of purpose related to reducing challenging behaviors versus facilitating the acquisition of adaptive skills. This characteristic was coded by searching for the terms “purpose,” “aim,” and “study,” and reviewing all instances of each to identify statements of purpose. If an article included a statement of purpose which incorporated terms such as “reduc...,” “treat...,” or “decreas...,” then the article was coded as having an aim related to behavior reduction. On the other hand, if an article included a statement of purpose which incorporated terms such as “teach...,” “train...,” “increas...,” or “acq...,” then the article was coded as having an aim related to skill acquisition. The coding of aims related to behavior reduction versus skill acquisition were not mutually exclusive; some articles included both types of aims and other articles included neither type of aim. In cases that were unclear, coders were instructed to read the entirety of the last paragraph of the introduction section and first paragraph of the discussion section prior to determining what to code.

**Fig. 1:**
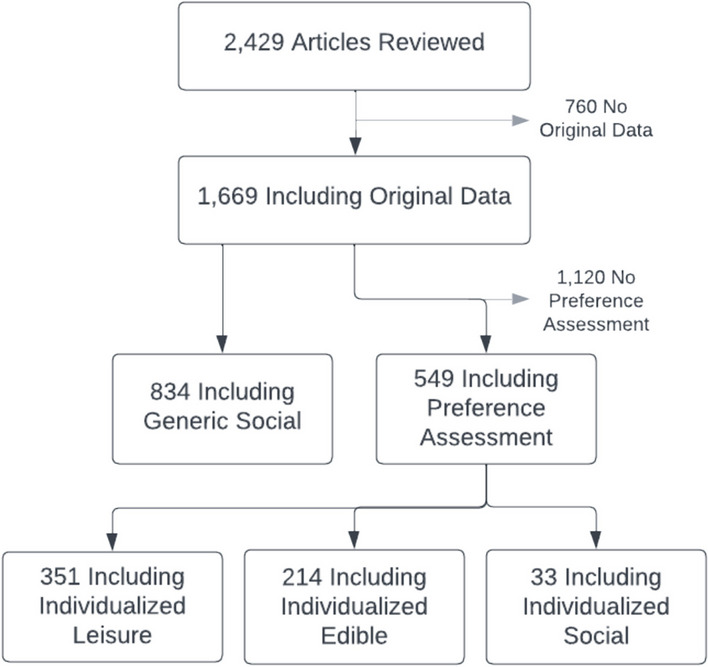
A Flowchart Summarizing the Review Process

To illustrate the coding procedures, the coding results for six articles included in the review that had been recently published in the *Journal of Applied Behavior Analysis* are provided online as “Supporting Information.” These examples were selected to illustrate the range of coding outcomes obtained across different articles. For example, many variables were not coded for some articles because they did not include original data or conduct a preference assessment, whereas all variables were coded for other articles which included original data, conducted a preference assessment, and described an aim other than evaluating or improving preference assessment methodology. Interested readers are encouraged to select one of the articles and apply the coding procedures above to gain a clearer conceptualization of the dependent measures included in this review. Figure 1 summarizes the review process, including the number of articles included and excluded at each step of the review process as well as the number of articles for which each variable was coded.

### Data Analysis

Following the completion of these five coding steps, we analyzed the resulting data in six ways. First, we calculated the percentage of articles per year that included original data by dividing the total number of articles including original data during a given year by the total number of articles during that year and multiplying the quotient by 100. Second, we calculated the percentage of articles per year in which a preference assessment was conducted by dividing the total number of articles including a preference assessment during a given year by the total number of articles including original data during that year and multiplying the quotient by 100. Third, we calculated the percentage of articles conducting a preference assessment that evaluated edible, leisure, or social stimuli by dividing the total number of articles including a preference assessment for each type of stimulus during a given year by the total number of articles conducting a preference assessment during that year and multiplying the quotient by 100. Fourth, we calculated the percentage of all articles including individualized social stimuli (i.e., social consequences based on the results of a preference assessment), generic or nonindividualized social stimuli (i.e., social consequences not based on the results of a preference assessment), or no programmed social stimuli by dividing the total number of articles meeting each of the above criteria during a given year by the total number of articles including original data during that year and multiplying the quotient by 100. Fifth, we evaluated the influence of different characteristics of each article’s purpose on the likelihood of evaluating edible, leisure, or social stimuli and the likelihood of programming individualized, generic, or no social stimuli as consequences. In particular, we divided the total number of articles evaluating a given stimulus type (i.e., edible, leisure, social) or incorporating different types of social stimuli (i.e., individualized, generic, or none) and meeting a given criteria for different purpose characteristics (i.e., preference assessment, behavior reduction, skill acquisition, intervention, or assessment focus) by the total number of articles that included a preference assessment.

### Intercoder Agreement

To evaluate intercoder agreement, a second coder independently coded all variables for 377 out of the 2,429 (16%) total articles and 116 of the 524 (22%) of articles that included original data and conducted a preference assessment. We randomly (i.e., using a random number generator) selected 3 articles from each year of each individual journal, which accounted for 135 of the 377 articles evaluated by a second coder. In addition, we randomly selected three years (i.e., 2017, 2018, 2022) of the *Journal of Applied Behavior Analysis* (i.e., the journal including the largest number of articles) for which to evaluate interobserver agreement for all articles published, which accounted for 194 of the total 377 articles evaluated by a second coder. We calculated interobserver agreement for a larger subset of articles published in the *Journal of Applied Behavior Analysis* solely because this was the first journal reviewed. For articles published in 2023, a second coder independently coded all variables for approximately 20% of the articles published in each journal, which accounted for 58 of the 377 of the total articles evaluated by a second coder.

Intercoder agreement was calculated by comparing observers’ data across each dependent measure (i.e., inclusion of original data, inclusion of preference assessment, type of stimulus evaluated, inclusion of any social consequences, and whether it included a statement of purpose specific to preference assessment, assessment strategies, intervention strategies, behavior reduction, or skill acquisitions) for each article. For each measure, an agreement was scored if both observers entered the exact same value (e.g., a “ + ” to indicate edible for reinforcer type, a “-” to indicate no edible for type of stimulus evaluated, or an “NA” if the coder did not code that variable for that article). A disagreement was scored if there was any discrepancy in the value recorded. All measures were compared for each article, regardless of whether the same number of measures were coded. Measures coded by one coder, but not the other, were automatically scored as disagreements. For example, if one coder indicated an article included original data and a preference assessment and therefore coded all variables, but the other indicated that the article did not include original data, then this constituted a disagreement on all measures (i.e., not just original data). The number of agreements was divided by the total number of dependent measures coded and then that quotient was multiplied by 100 to obtain the intercoder agreement score. Table 1 presents the intercoder agreement scores for all coded variables, intercoder agreement averaged above 95% for all variables and years. The authors reviewed all remaining disagreements to come to a consensus on what should be coded.

**Table 1:** Summary of Intercoder Agreement across All Coded Variables

Variable	Mean Intercoder Agreement (Range across Years)
Original Data	97.9% (95% to 100%)
Preference Assessment	96.8% (96% to 99%)
Stimulus Type	98.1% (97% to 99%)
Generic Social Stimuli	95.2% (93% to 98%)
Article Purpose Characteristics	98.3% (97% to 100%)
All Variables	97.7% (96% to 99%)

## Results

**Fig. 2:**
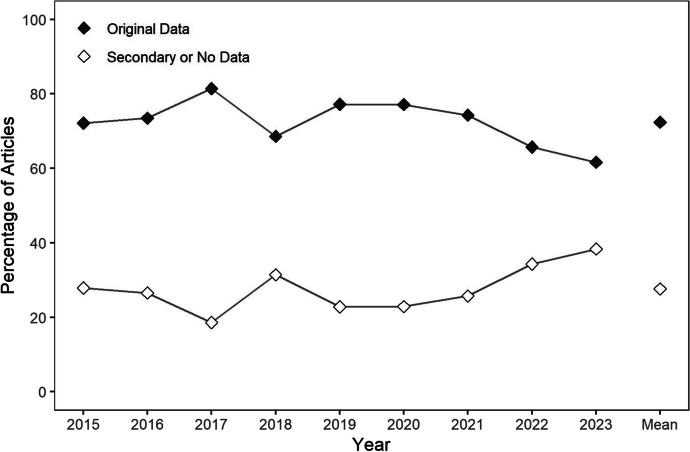
The Percentage of Articles including Original Data

The next four figures depict the percentage of articles (y-axis) across years (x-axis) for which different characteristics (e.g., conducted a preference assessment) were coded. The disconnected data points on the far right of the x-axis represent the mean percentage of articles across years. A legend indicating the meaning of the fill of the data points for each figure is provided in the upper left. The first step of the review was to evaluate each of the 2,429 articles included to determine whether they included original data, meaning that it was not a discussion or commentary and did not constitute a review, synthesis, or secondary analysis of previously published data. Figure 2 depicts the results of this evaluation. Across years, articles were much more likely to include original data rather than no data or secondary analyses of previously published data. Considering all years, 72% of articles included original data, whereas 28% of articles included no data or secondary analyses of previously published data. However, beginning in the year 2019, a decreasing trend is evident in the percentage of articles including original data.

**Fig. 3:**
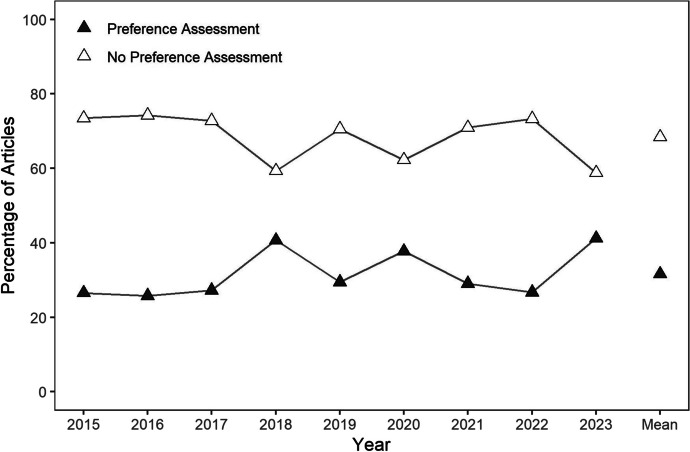
The Percentage of Articles including a Preference Assessment

For the 1,669 articles including original data, the next step of the review was to evaluate whether they described individualizing their reinforcement procedures by conducting some form of stimulus preference assessment. Figure 3 depicts the results of this evaluation. Across years, a minority of articles described conducting a preferences assessment, but authors described including a preference assessment in at least 25% of articles each year. Considering all years, 32% of articles described including a preference assessment, whereas 68% of articles did not specify that a preference assessment was conducted. No clear trends were evident in the percentage of articles describing that some form of stimulus preference assessment was included. We did not code preference assessment type or modality, but a wide variety of different stimulus preference assessment methods (e.g., single-stimulus, paired-stimulus, multiple stimulus without replacement, free operant, social interaction preference assessment) and modalities (e.g., actual items, picture-based, video-based, vocally mediated) were described.

**Fig. 4:**
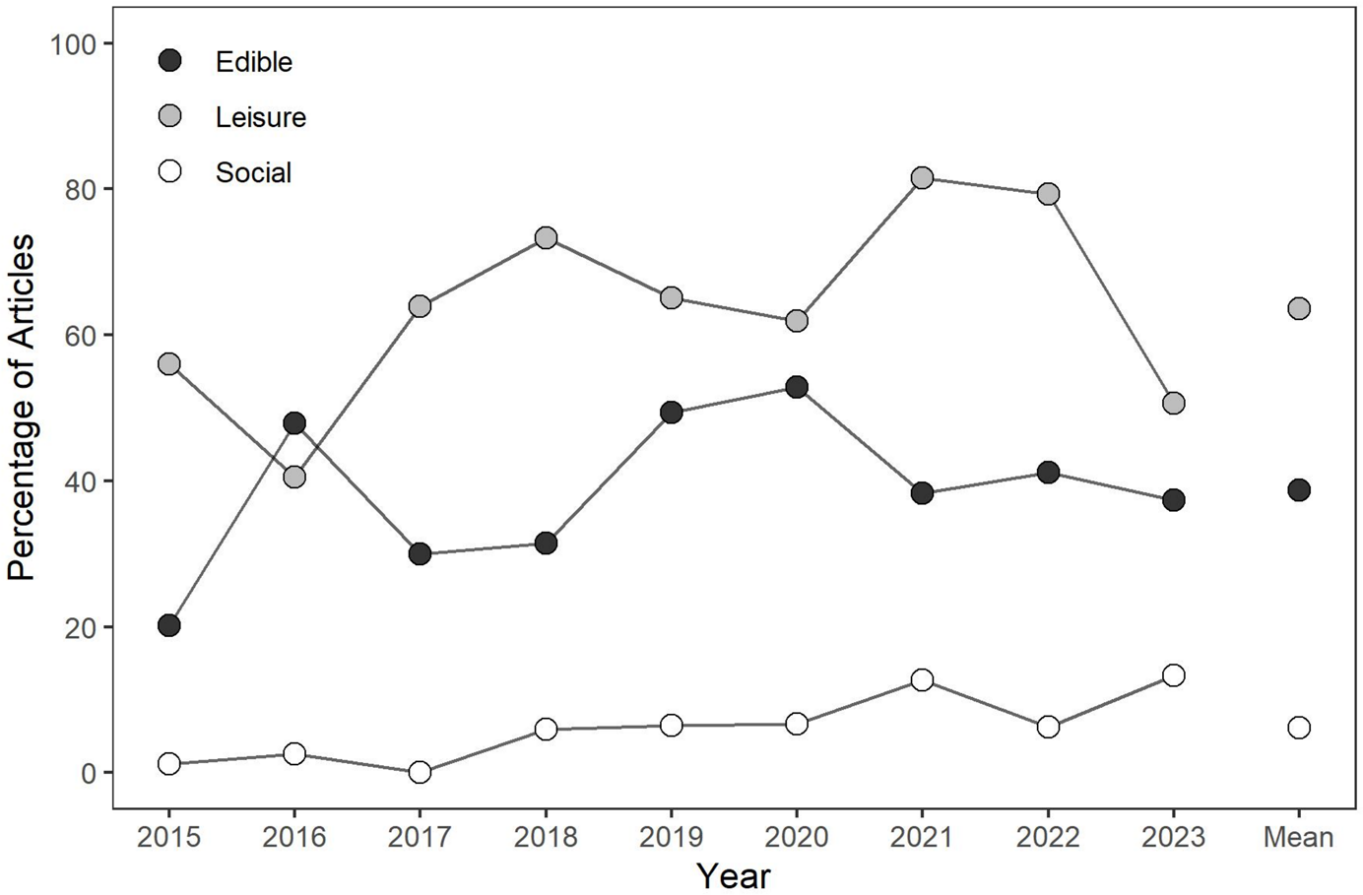
The Percentage of Articles Utilizing Individualized Edible, Leisure, and Social Reinforcers

For the 549 articles that included both original data and described including some form of stimulus preference assessment, the next step of the review was to evaluate the percentage of articles using individualized edible, leisure, or social reinforcers. Figure 4 depicts the results of this evaluation. Across most years, the majority of articles described using preference assessments to incorporate individualized leisure stimuli, articles were slightly less likely to describe incorporating individualized edible stimuli, and relatively few articles described incorporating individualized social stimuli. Considering all years, authors described evaluating individualized leisure stimuli in 64% of articles, individualized edible stimuli in 39% of articles, and individualized social stimuli in 6% of articles. A clear increasing trend is evident in the percentage of articles that described incorporating individualized leisure stimuli, whereas less pronounced increasing trend is evident in the percentage of articles that described including social stimuli. The presence of increasing trends across multiple stimulus types may indicate an increase in the percentage of articles that describe incorporating multiple types of individualized stimuli as consequences.

**Fig. 5:**
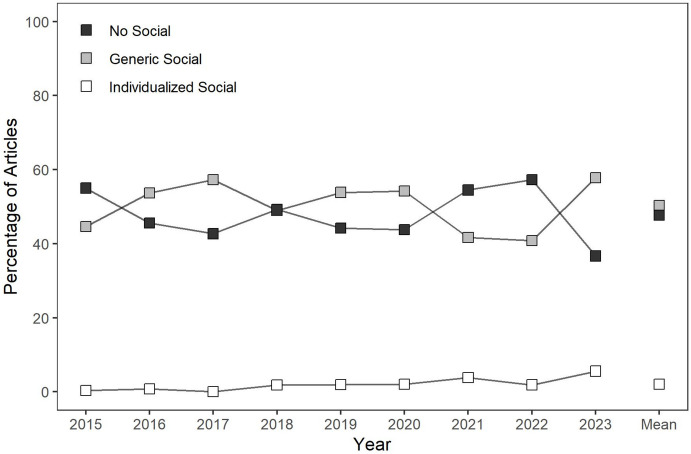
The Percentage of Articles including Individualized Social Stimuli, Generic Social Stimuli, or No Social Stimuli

For all 1,669 articles including original data, the next step of the review was to evaluate whether each article described including any form of social stimuli as programmed consequences. In particular, we evaluated the percentage of articles that described including individualized social stimuli, generic or nonindividualized social stimuli, or no programmed social consequences as a part of their procedures. Figure 5 depicts the results of this evaluation. When compared to all articles including original data (i.e., not just those conducting a preference assessment as in Fig. 4), an even smaller minority of articles included some form of individualized social stimuli (i.e., always less than 5% of articles per year), whereas a similar percentage of articles included generic social stimuli and no social stimuli across years. Considering all years, 2% of articles described incorporating individualized social stimuli, 50% of articles included generic social stimuli, and 48% of articles included no programmed social stimuli as consequences. No clear trends across years were evident in these data.

**Fig. 6:**
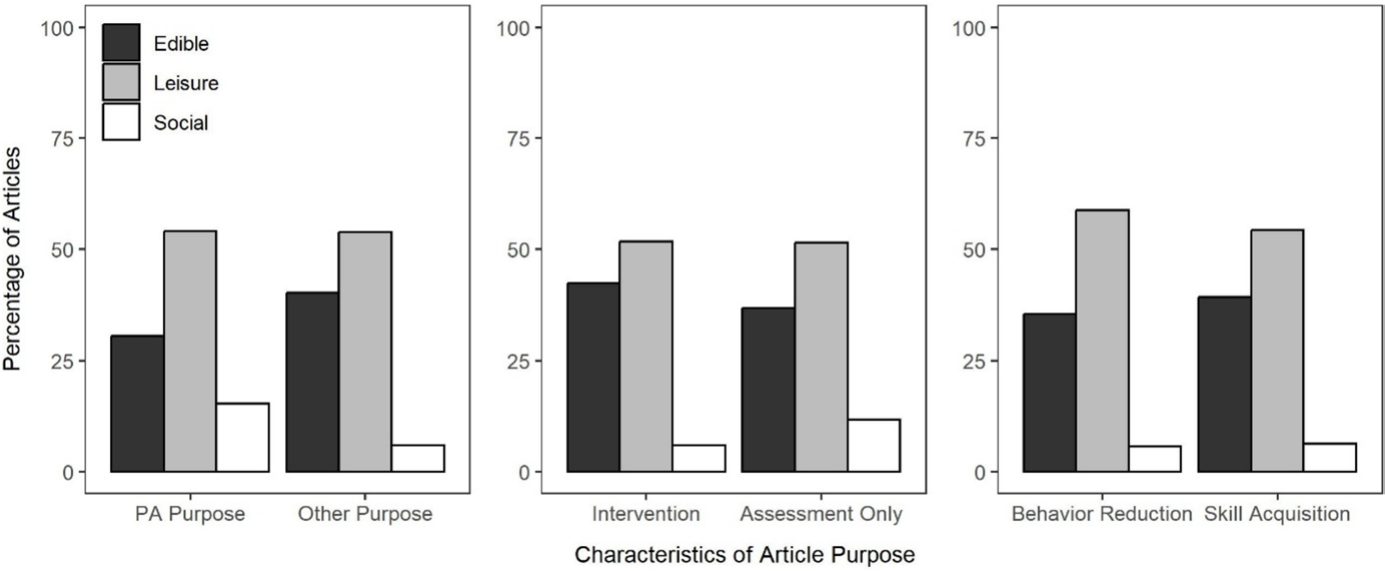
The Percentage of Articles with Different Purpose Characteristics that included Individualized Edible, Leisure, and Social Stimuli

One limitation of the preceding analyses is that they aggregated all articles reviewed, regardless of heterogeneity in the purpose of the articles. To address this limitation, we evaluated how the relative likelihood with which articles described evaluating individualized edible, leisure, or social stimuli and using individualized, generic, or no social stimuli varied across different characteristics of the purpose stated in each article. For the 549 articles that included both original data and some form of stimulus preference assessment, we coded a series of dichotomous characteristics from their statement of purpose: (1) whether or not their purpose was specific to evaluating and improving methods of stimulus preference assessments; (2) whether their statement of purpose described interventions to facilitate behavior change or only aims related to assessing behavior; and (3) whether their statement of purpose described aims related to behavior reduction or skill acquisition. Figure 6 depicts the relation among these variables and the type of stimuli evaluated in the preference assessment. The x-axis depicts the characteristics of the statement of purpose and the y-axis depicts the percentage of articles evaluating each of the type of stimuli, as denoted by the different-colored bars. For the most part, this analysis suggests that these characteristics of the article’s purpose exerted little influence on the relative likelihood of evaluating different types of stimuli. However, a few small differences are worth noting. Articles that described their purpose in terms of evaluating or improving stimulus preference assessment methodology were slightly more likely to evaluate social stimuli and slightly less likely to evaluate edible stimuli. The same was true for articles that only assessed behavior relative to those that evaluated interventions designed to facilitate behavior change. Finally, articles that aimed to facilitate skill acquisition rather than behavior reduction were slightly less likely to evaluate leisure stimuli and slightly more likely to evaluate edible stimuli.

**Fig. 7:**
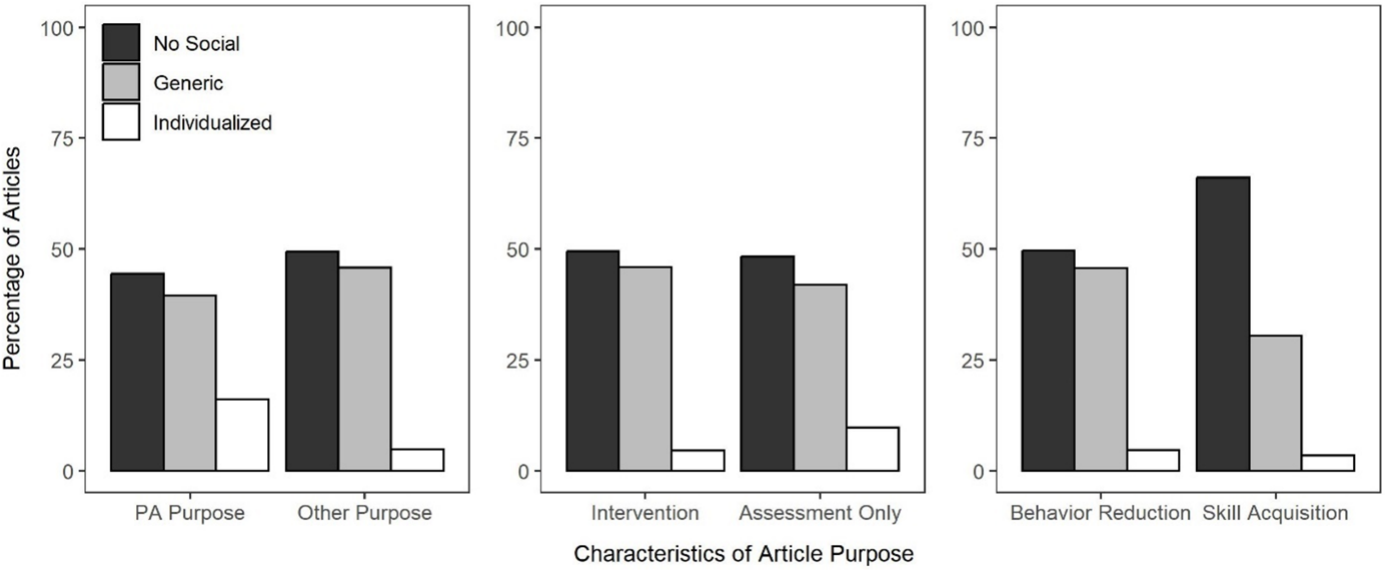
The Percentage of Articles with Different Purpose Characteristics that included Individualized Social Stimuli, Generic Social Stimuli, or No Social Stimuli

Figure 7 depicts the relation between these purpose variables and the type of social stimuli incorporated as consequences within each article. Again, it seems the evaluated characteristics of the article’s purpose exerted minimal influence on the relative likelihood that individualized, generic, or no social stimuli were included. However, some of the same differences apparent in Fig. 6 are also apparent here: when the purpose was to improve preference assessment methodology or only to assess rather than change behavior, articles were more likely to incorporate individualized social stimuli. The most salient influence of these variables was that articles that aimed to facilitate skill acquisition were more likely to include no social stimuli as programmed consequences and less likely to incorporate generic social stimuli as consequences compared to articles that aimed to facilitate behavior reduction.

## Discussion

This review provides an evaluation of the identification and use of social versus nonsocial reinforcers in recent applied, behavior-analytic research. Our results suggest that most articles included original data (*M* = 72%) and that many of those described conducting preference assessments to individualize their reinforcement procedures (*M* = 32%). When individualizing reinforcement procedures using stimulus preference assessments, articles were most likely to describe using leisure reinforcers (*M* = 64%) and second-most likely to describe using edible reinforcers (*M* = 39%). Articles were much less likely to describe using individualized social reinforcers (*M* = 6%). Moreover, articles often did not describe incorporating social stimuli as programmed consequences (*M* = 48%) and when they did, they were often generic and not individualized (*M* = 50%). The analyzed characteristics of the stated purpose of the article only exerted a small influence on the likelihood with which different types of reinforcers were evaluated or different types of social stimuli were employed as programmed consequences.

Juxtaposition of these results with those of previous descriptions of research and clinical practices related to reinforcer identification may prove useful. The current review focused on the identification and use of different types of reinforcers across a wide spectrum of recent applied behavior-analytic research. However, recent reviews of reinforcement practices in research related to skill acquisition interventions provide some relevant points of comparison. As in the current review, Frank-Crawford et al. (2024) found that more studies employed nonsocial rather than social reinforcers. In particular, they found that 58% of studies evaluating discrete trial instruction procedures incorporated edible or leisure stimuli, whereas 21% of studies incorporated social stimuli in their programmed reinforcement contingencies. Both Weinzstok et al. (2023) and Frank-Crawford et al. found skill acquisition procedures to be more efficacious when nonsocial stimuli (e.g., leisure or edible items, tokens) rather than social stimuli were programmed as reinforcers. However, as in the current review, they found that social stimuli were almost always generic and rarely individualized based on the results of a preference assessment. Taken together, the results of the current study and related previous reviews suggest that independently and comparatively evaluating *individualized* social reinforcers is an important direction for future research.

Regarding clinical practices, Morris et al. (2024) asked practicing behavior analysts whether they used different types of reinforcers and found that most clinicians reported employ all three types of reinforcers to some extent, with fewer at least sometimes using edible than leisure or social reinforcers. Beyond whether or not different types of reinforcers are employed, they also asked respondents about their relative likelihood of using different types of reinforcers and what types of social reinforcers respondents employed. They found that respondents reported most frequently using leisure reinforcers, followed by social reinforcers, and then edible reinforcers in clinical practice and that social reinforcers, when employed, were sometimes generic but usually individualized. Some important similarities and differences are evident between these reported clinical practices and our review of research practices. In both research and clinical practice, a bias in favor of nonsocial reinforcers is evident and, when social reinforcers are used, they are not always individualized. However, relative to reported clinical practices, our review suggests that behavior-analytic researchers are more likely to use individualized edible reinforcers and less likely to use individualized social reinforcers.

Reliance on edible reinforcers and infrequent use of individualized social reinforcers in research may be concerning for a few reasons. First, reliance on edible reinforcers may have deleterious effects on the health and well-being of individuals participating in the research as well as those receiving services based on that research (see Kronfli et al., 2020, for a related discussion). Second, the limited use of social reinforcers may prevent researchers, their participants, and participants’ support system (e.g., caregivers, teachers) from contacting the benefits of social reinforcers. Third, and perhaps most important, reliance on edible reinforcers and limited use of social reinforcers may inhibit the social or ecological validity, dissemination, and adoption of behavior-analytic research and corresponding services. This problem can be conceptualized within the framework of consumer behavior analysis (e.g., Foxall, 1987, 2016): behavior analytic researchers have focused on the efficacy of interventions (i.e., utilitarian reinforcers), while neglecting differential social contingencies related to implementing different intervention strategies (i.e., informational reinforcers). Such informational reinforcers may be equally, if not more, important determinants of whether an intervention will be adopted (e.g., Gilroy & Picardo, 2022).

Our findings only describe how different types of reinforcers have been used in recent behavior-analytic research. This description may inform and provide the impetus for subsequent experimental questions that could elucidate whether and how the relative likelihood of using different types of reinforcers should change. Before an increased use of individualized social reinforcers can be recommended, future research should demonstrate the utility of social reinforcers by utilizing them to replicate and extend well-documented intervention effects that have been obtained with nonsocial reinforcers. For example, researchers have shown that the use of positive, nonsocial reinforcers can produce larger and more durable effects than negative reinforcement (i.e., escape from instruction) for behavior maintained by negative reinforcement (e.g., Bonner et al., 2022; Slocum & Vollmer, 2015), but it is unclear if individualized social reinforcers may afford the same benefits (although, see Scheithauer et al., 2022, for a demonstration). Likewise, researchers have found that established nonsocial reinforcers can be effective at establishing praise (e.g., Dozier et al., 2012; Dudley et al., 2019; Jimenez-Gomez et al., 2024; Taylor-Santa et al., 2014) or speech sounds (e.g., Esch et al., 2009; Lepper et al., 2017) as conditioned reinforcers, but it is unclear if established social reinforcers could be just as, if not more, effective in this context (although, see Participant Jack in Axe & Laprime, 2017, for a demonstration). It also remains unclear how the use of different types of reinforcers may indirectly influence social behavior. Future research could utilize assessments of sociability (e.g., Call et al., 2013; Morris & Vollmer, 2020d, 2021, 2022b; Morris & Pizzuto, 2023) to evaluate how experience with interventions using social versus nonsocial reinforcers may indirectly influence the function of social interaction in general, the discriminative function of others’ behavior, and the occurrence of related social communication and leisure skills.

Beyond evaluating the utility of individualized social reinforcers, it will also be important for future research to compare relative efficacy and acceptability of interventions incorporating social versus nonsocial reinforcers. Researchers have previously compared the relative efficacy of social and nonsocial stimuli in the context of reinforcer assessments (Clay et al., 2018) and skill acquisition (Leaf et al., 2014); however, more research is needed to understand the relative utility of different types of reinforcers. For example, it remains unclear how the effects on different proportions of social to nonsocial reinforcers (i.e., not just all of one or the other) may affect intervention efficacy, generalization and maintenance of intervention effects, and acceptability measures (e.g., social validity, adoption). Researchers should pursue these questions through direct experimentation (e.g., manipulating reinforcer type and observing its effects) as well as ecologically oriented questions about how factors such as reinforcer type and the size or immediacy of intervention effects influence interested parties’ (e.g., caregivers, teachers, policy makers) consumption or adoption of intervention strategies (e.g., Call et al., 2015a, 2015b; Gilroy & Feck, 2022).

The current study also has several limitations that suggest additional directions for future research. Our analysis of research practices related to reinforcer type was somewhat shallow and was constrained by utilizing the inclusion of preference assessments as a criterion for whether a study employed individualized reinforcers. Related to this, coding these variables at the participant level rather than the article level may have allowed for more precise conclusions, given that the types of reinforcers and methods of evaluation sometimes vary across participants within the same study. In future research, it may be useful to include more detailed measures such as preference assessment type, modality, and function or use of the preference assessment results.

The mechanisms underlying the relative likelihood of using different types of reinforcers remain unclear and are an important area for future descriptive and empirical research. We included a coarse analysis of characteristics of each article’s purpose, which may have imperfectly categorized the purpose of each article. For example, it is possible that some articles may have included original data, described conducting a preference assessment, and included “train...” in their statement of purpose that were not actually skill acquisition studies. A more in-depth analysis of additional purpose characteristics for the full sample of articles included may allow for useful additional insights. In future research, it could be beneficial to evaluate how reinforcer type and the degree of individualization vary based on specific article key words or participant-level variables like age, diagnoses, or specific target behaviors.

As noted in the introduction, our categorization of reinforcement types was nonexhaustive. For example, we did not account for the use of tokens (e.g., Hackenberg, 2018; Leon et al., 2016) or the delivery of reinforcers in distributed versus accumulated formats (e.g., DeLeon et al., 2014; Frank-Crawford et al., 2021). These factors may just as strongly affect the efficacy and acceptability of interventions as reinforcer type and should be incorporated into future research. Likewise, focusing on preference assessments as the method of individualization made completing a review of this magnitude feasible, but it may also be useful to incorporate other methods of individualizing reinforcers, such as caregiver report (e.g., Fisher et al., 1996). Even though such methods are unlikely to correspond to more rigorous assessments (e.g., Cote et al., 2007; Morris & Vollmer, 2022c), they still constitute individualization and can result in the identification of stimuli that function as reinforcers (Morris & Vollmer, 2022c).

In summary, our results demonstrate that behavior-analytic researchers often rely on nonsocial reinforcers and suggest that we may not be taking advantage of the benefits that individualized social reinforcers may afford. In some cases, there is clear rationale for the type of reinforcer used (e.g., functional reinforcers to reduce and replace challenging behavior), but, in other cases, different types of reinforcers may be equally suitable (e.g., skill acquisition, establishing new reinforcers, non-function-based interventions). In these cases, DeLeon et al.’s (2013) decision-making framework may serve as a useful guide. First, use or establish social reinforcers prior to employing nonsocial reinforcers. Second, only if sufficient social reinforcers cannot be identified or established, use accumulated leisure reinforcers in a token system or distributed, direct leisure reinforcers if a token system is infeasible. Third, only if sufficient leisure reinforcers cannot be identified or established, use accumulated edible reinforcers in a token system or distributed, direct edible reinforcers if a token system is infeasible. Going forward, we should consider applying contingencies to support adherence to this framework; doing so has the potential to improve the efficacy, acceptability, and adoption of applied behavior analysis across the research-practice continuum.

## Supplementary Information

Below is the link to the electronic supplementary material.

Supplementary file1 (DOCX 21 KB)

## Data Availability

The current study is a review of previously published data, but additional information and analyses are available from the first author upon reasonable request.

## Footnotes

1 It is important to note that this a nonexhaustive categorization of potential artificial programmed reinforcers. The way that the omission of some common categories (e.g., tokens) limits our results are included in the “Discussion.”.

2 In addition, social reinforcers may be one of relatively few consequences (e.g., music) that are well suited for use in the context of synchronous reinforcement intervention (e.g., Diaz de Villegas et al., 2020; Hardesty et al., 2023).

3 To improve concision throughout the method section, we use the term “search” as a shorthand for the process of using the Cntrl + F function to identify all occurrences of a specific word or phrase in a document. In all cases, the exact word input into the search box is presented in quotation marks. In some cases, only partial words were searched (e.g., “pref...”) to ensure all relevant variations could be captured (e.g., “preferred” or “preference”).

4 It is important to note that this coding scheme relies solely on the authors’ use of the term “preference assessment.” The term consistently appeared as a reference to previously published methods of stimulus preference assessment (e.g., DeLeon et al., 1996; Fisher et al., 1992), but we did not discriminate or exclude instances where it was misapplied to describe less formal (e.g., pretrial choice) or indirect (e.g., asking the participant or their caregiver) methods.
