# Supplementary material for: On the Identification and Use of Social versus Nonsocial Reinforcers: A Review of Research Practices
Source: Perspect Behav Sci. 2024 Oct 29;47(4):739–61. doi: 10.1007/s40614-024-00426-0 (PMC11582244; doi:10.1007/s40614-024-00426-0)
Supplement: Supplementary file 1 — Supplementary file1 (DOCX 21 KB) [file 40614_2024_426_MOESM1_ESM.docx]

**Supporting Information**

***Key****:*

+ = criteria were met to code this variable as present

- = criteria were met to code this variable as absent, or criteria were not met to code this variable as present

grey background = variable was coded

white background = variables was not coded

|  |  |  | Stimulus Type | | |  | Purpose Characteristics | | | | |
| --- | --- | --- | --- | --- | --- | --- | --- | --- | --- | --- | --- |
| Title of Article | Original Data | Conducted SPA | Edible | Leisure | Social | Any Social | Evaluate/  Improve SPA | Intervention | Assessment | Behavior Reduction | Skill Acquisition |
| Procedural parameters in equivalence-based instruction with individuals diagnosed with autism: A call for systematic research.  <https://doi.org/10.1002/jaba.998> | *-* |  |  |  |  |  |  |  |  |  |  |
| Telehealth replication of the trial-based ongoing visual-inspection criteria.  <https://doi.org/10.1002/jaba.994> | *+* | *-* |  |  |  | *+* |  |  |  |  |  |
| An evaluation of preference stability within MSWO preference assessments for children with autism. <https://doi.org/10.1002/jaba.988> | *+* | *+* | *+* | *+* | *-* | *-* | *+* |  |  |  |  |
| Increasing young children's honest reports and decreasing their transgressions.  <https://doi.org/10.1002/jaba.960> | *+* | *+* | *+* | *+* | *-* | *+* | *-* | *+* | *-* | *+* | *+* |
| A comparison of synchronous and noncontingent stimulus delivery on task engagement  <https://doi.org/10.1002/jaba.986> | *+* | *+* | *-* | *-* | *-* | *-* | *-* | *+* | *-* | *-* | *+* |
| Increasing social time allocation and concomitant effects on mands, item engagement, and rigid or repetitive behavior. <https://doi.org/10.1002/jaba.919> | *+* | *+* | *-* | *-* | *+* | *+* | *-* | *+* | *-* | *-* | *+* |
